# Evaluation of the Prevalence of Chronic Kidney Disease and Rates of Oral Antidiabetic Prescribing in Accordance with Guidelines and Manufacturer Recommendations in Type 2 Diabetic Patients within a Long-Term Care Setting

**DOI:** 10.1155/2014/151706

**Published:** 2014-02-25

**Authors:** Ning Wu, Xia Yu, Mallik Greene, Gary Oderda

**Affiliations:** ^1^Health Economics and Epidemiology, Evidera, 430 Bedford Street, Suite 300, Lexington, MA 02420, USA; ^2^Boehringer Ingelheim Pharmaceuticals, Inc., 900 Ridgebury Road, Ridgefield, CT 06877, USA; ^3^University of Utah School of Pharmacy, 30 South 2000 East, Salt Lake City, UT 84112, USA

## Abstract

This retrospective study assessed the prevalence of moderate to severe chronic kidney disease (CKD) among nursing home (NH) residents with type 2 diabetes. The pattern of oral antidiabetic drug (OAD) use and their concordance with the National Kidney Foundation (NKF) guideline and prescribing information (PI) was also assessed. About half (47%) of diabetic residents had moderate to severe CKD. A little over a quarter of the 186 residents using OADs received at least one NKF-discordant OAD prescription. Metformin was the most commonly misused OAD. PI nonconcordance was observed in 58.6% of residents and was highest in glipizide and metformin users. With the high prevalence of moderate to severe CKD in NH residents with diabetes, physicians should consider residents' renal function when choosing treatment plans and review treatments regularly to check compliance with the NKF guidelines or PIs.

## 1. Introduction

Diabetes is a public health concern of epidemic significance, affecting an estimated 25.8 million people in the United States (US) [1]. Approximately one-quarter of the 1.3 million nursing home (NH) residents in the US have diabetes [2, 3]. Diabetes is also associated with a significant economic burden. In 2007, the estimated total direct and indirect annual costs of the disease were $174 billion in the US [1, 4]. In the same year, 32% of Medicare expenses incurred within long-term care (LTC) settings were for diabetes care [5]. The diabetes-related costs in the LTC settings also increased rapidly. The estimated cost of care for diabetic residents within LTC facilities increased from $13.9 billion in 2002 to $18.5 billion in 2007 [5].

Chronic kidney disease (CKD) is a common complication of diabetes; it contributes to clinical complexity of diabetes management and the economic burden of diabetes in the LTC settings. According to the Centers for Disease Control and Prevention (CDC), up to 44% of new cases with end-stage renal disease (ESRD), the most severe form of CKD, were attributable to diabetes in 2008 [6]. A survey of the NH population reported that 34% of surveyed diabetic NH residents also had CKD [7].

Diabetic patients with renal impairment are clinically complex and vulnerable to drug-drug interactions and adverse events associated with medication [7]. Therefore, it is important to assess their clinical characteristics and the treatments they received. NH residents with diabetes often have multiple comorbidities and receive multiple medications [3, 8, 9]. Meanwhile, dementia and functional disability, which are common among NH residents, make it difficult to manage residents' drug therapy [10, 11]. Diabetic NH residents with CKD are even more vulnerable to drug-drug interaction or severe adverse events than those without CKD. Impaired kidney function may lead to a longer biologic or elimination half-life and increases the risk of drug-drug interactions or severe adverse events [12, 13]. Some medications or combinations of medication use may be safe in individuals with normal kidney function, but can be dangerous in individuals with CKD. However, there is limited information on the treatment patterns and polypharmacy among NH residents with diabetes and renal impairment [3]. Using a nationally representative sample, Dwyer et al. reported that over 40% of NH populations received 9 or more medications in one calendar year [14, 15]. No existing studies have assessed the prevalence of polypharmacy or type of medications received among NH residents with both diabetes and CKD.

The National Kidney Foundation (NKF) published clinical practice guidelines on the management of diabetes in patients with CKD [16]. These guidelines recommend that the prescribed dosage of several oral antidiabetic drugs (OADs) should be altered or avoided in patients with diabetes and comorbid CKD [16]. A recent study found that OAD treatment not concordant with NKF guideline recommendations led to worse clinical and economic outcomes [17]. Despite a large body of literature on diabetic patients with CKD, published data on how diabetes is treated in LTC residents are limited [18, 19]. The objective of this retrospective database study was to assess the prevalence of CKD as well as to compare the use of OAD medication with NKF guidelines and drug package insert (PI) recommendations among NH residents with diabetes.

## 2. Materials and Methods

### 2.1. Study Database

The data were extracted from the AnalytiCare LTC database from 2008 to 2011. The AnalytiCare LTC database provides integrated LTC data comprised of demographic data, pharmacy claims, and clinical assessment data (also known as the minimum data set [MDS]) of more than 100,000 residents in NHs from 4 US geographical regions, with laboratory results available for about 10% of the sample. Since NH residents' laboratory results were necessary to confirm the diagnosis and severity of CKD, all NH residents with LTC data along with laboratory results were included in this study analysis.

The demographic files contained information about resident's age, gender, race/ethnicity, Medicare/Medicaid eligibility, level of education, marital status, and state of residence. The pharmacy claims contained details of medications dispensed to the residents during their NH stays, which included the national drug code (NDC) number, dispensing date, quantity dispensed, and days of supply. The laboratory test records include test name, normal ranges, and test dates and readings. The MDS was collected as part of the federally mandated process for improving quality of care in Medicare/Medicaid certified NHs. The process requires comprehensive clinical assessments of each resident upon NH admission, quarterly, annually, at discharge, and at any significant change in health status. To fill out the MDS forms, nurses or MDS coordinators in each nursing home conduct chart review, resident assessment, and caregiver interviews to gather information on resident clinical and functional status and resource utilization during most recent 7- or 14-day period.

### 2.2. CKD Prevalence Estimate in Residents with Diabetes

The prevalence of CKD in diabetic residents in NHs was estimated for each calendar year based on the data from 2008 to 2011. Residents were included as the denominator of the prevalence estimation of a calendar year if they met the following criteria: (1) continuously stayed in the NH for at least 30 days during the year, (2) were at least 18 years old on the day of the first MDS assessment during 2008–2011, (3) had at least 1 MDS assessment with MDS item I2900 suggesting diabetes, (4) and had laboratory data during the calendar year. Residents with mental retardation and/or developmental disabilities due to organic conditions, that is, diseases caused by a dysfunction of an organ or enzyme system, rather than psychiatric or functional conditions, were excluded from the study. Examples of conditions for exclusion include Down syndrome, autism, and epilepsy.

Each year, residents with moderate to severe CKD were identified via both MDS and laboratory results. Moderate to severe CKD was defined as at least 1 MDS assessment suggesting renal impairment, ESRD, or dialysis; by International Classification of Diseases, 9th Revision, Clinical Modification (ICD-9-CM) diagnosis codes 585.3 (moderate CKD), 585.4 (severe CKD), 585.5 (very severe CKD), or 585.6 (ESRD) (Table 1); or by estimated glomerular filtration rate (eGFR) <60 mL/min/1.73 m^2^. In the MDS, ICD-9-CM diagnosis fields were designed to capture conditions not included in the comprehensive diagnosis checklist in MDS Section I. Thus, it was expected that a small number of CKD patients would be identified via diagnosis codes, and the majority of residents would be identified via lab results. CKD stage was also identified through laboratory results based on the NKF definition of CKD using eGFR. The prevalence (per 100 patients) of CKD in diabetics was determined by the formula given below:

(1) Prevalence  of  CKD  in  diabetics=Number  of  diabetes  patients  with  CKD  during  the  calendar  yearNumber  of  diabetic  residents  during  the  calendar  year×100.

### 2.3. Characteristics of Residents with and without Moderate and Severe CKD

To describe demographic and clinical characteristics and medication use of residents with diabetes, a subgroup of diabetic residents with a minimum of 90-day continuous stay during 2008–2011 and an eGRF test dated within 1 year before the continuous stay were selected from the residents identified to estimate prevalence. The 90-day continuous enrollment allowed us to assess the pattern of medication use during residents' NH stay. Demographic characteristics such as age, gender, race/ethnicity, and state of residence were assessed based on the MDS assessments that were conducted closest to the beginning of the 90-day assessment period. Clinical characteristics including health conditions and functional status, body mass index (BMI), and smoking status were also assessed based on the MDS. The number of unique medications that residents received during the 90-day period was assessed and presented as both continuous and categorical measures (i.e., 1–4, 5–9, 10–14, and 15+ unique medications). The proportion of residents with polypharmacy was calculated, and polypharmacy was defined as taking 9 or more unique medications [14, 20] during the 90-day period. Use of selected classes of medications was also reported. The proportion of residents receiving any antidiabetic treatment, OADs or glucagon-like peptide-1 agonists (GLP-1), insulin, and individual OADs were calculated. The descriptive analysis was further stratified by whether a resident had moderate to severe CKD (also referred to as stage 3–5 CKD).

### 2.4. Assessment of the Concordance of OAD Utilization with the NKF Guidelines and PIs

Concordance of OAD utilization with the NKF guidelines and PIs was assessed among residents with stage 3–5 CKD who received at least 1 OAD prescription mentioned in the NKF guidelines (Table 2). Because NKF requires changes in OAD regimen in residents with reduced eGF/R, [15] the analysis was restricted to residents with stage 3–5 CKD. The NKF guidelines do not include saxagliptin or linagliptin because the guidelines were published prior to their approval (saxagliptin was approved in 2009 and linagliptin approved in 2011 in the US). Therefore, the use of saxagliptin and linagliptin was only assessed according to PIs.

Residents were grouped into NKF-concordant and NKF nonconcordant cohorts by assessing their pharmacy claims against the NKF guidelines. Concordance was defined as residents who received an appropriate dosage of OADs, as suggested by the guidelines, and did not receive OADs that should be avoided. Since one resident may have received multiple OADs during the 90-day assessment period, each use was assessed against the NKF guidelines. Residents were classified as concordant if all the OADs dispensed during the 90 days were concordant with NKF guidelines. Residents were defined as nonconcordant if they received 1 or more prescriptions that were not concordant to recommendations. Similar methods were used to classify the medication use and patients into PI concordant and nonconcordant groups by comparing them against the PIs (Table 3).

### 2.5. Data Analyses

Descriptive analysis was conducted for residents with diabetes, stratified by the presence of moderate to severe CKD, and for NKF- and PI-concordant and nonconcordant cohorts, respectively. For continuous variables and counts (i.e., the number of medications), mean and standard deviations (SD) were reported. For categorical variables, frequency distributions with percentages were reported. To detect the statistically significant difference between concordant and nonconcordant cohorts, Student's *t*-tests were used for continuous variables, Fisher's exact tests were used for categorical variables, and Wilcoxon rank-sum test was used for count measures, such as number of unique medications received. *P* values below 0.05 were considered statistically significant. All analyses were performed using SAS version 9.2 (SAS, Inc., Cary, NC) statistical software.

## 3. Results

### 3.1. Prevalence of Moderate to Severe CKD among Diabetic Residents

**Figure 1:**
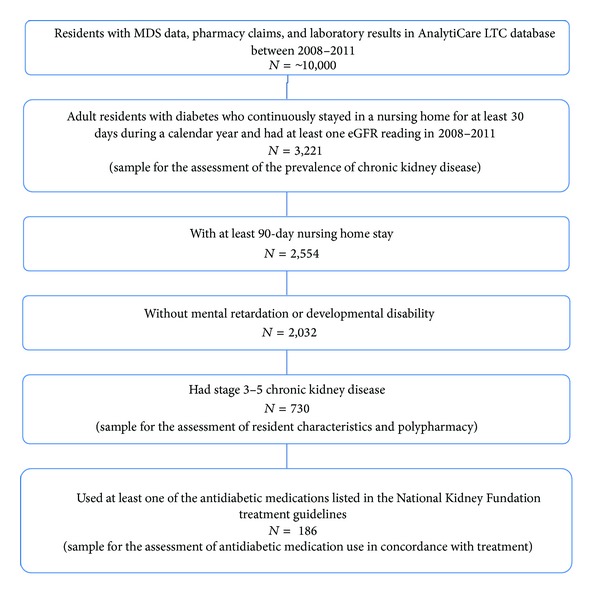
Sample selection.

**Figure 2:**
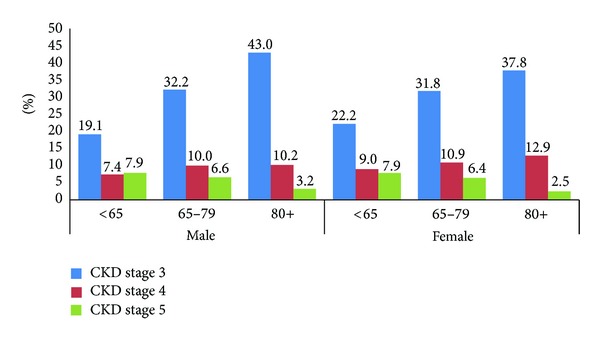
Prevalence of CKD by age and gender in nursing home residents with diabetes, in 2008–2011.

Of the 3,221 diabetic NH residents with at least 30-day nursing home stay and one eGFR value in 2008–2011 (Figure 1), approximately half of the diabetic NH residents (47.4%) (*n* = 1, 527) had moderate to severe CKD based on eGFR values, whereas only 18.2% were identified as having renal insufficiency/renal failure in MDS. The most common severity of CKD was stage 3 (31.4%), followed by stage 4 (10.4%) and stage 5 (5.6%). The prevalence of stage 3–5 CKD increased with age in both males (from 34.5% in <65 years old to 55.5% in ≥80 years old, *P* < 0.01) and females (from 39.0% in <65 years old to 53.2% in ≥80 years old, *P* < 0.01) (Figure 2). The prevalence of stage 3–5 CKD was not significantly different between male and female residents within each age group. Because the prevalence of CKD was similar across the calendar years, pooled results for 2008–2011 were presented.

### 3.2. Characteristics of Diabetic Residents with Moderate to Severe CKD

Of the 2,554 NH residents with diabetes and at least a 90-day continuous NH stay between 2008 and 2011, 91 were excluded because they resided in NHs due to mental retardation or developmental disability, and 431 were excluded because they did not have an eGFR test within 1 year before the 90-day continuous stay for the assessment of CKD stage. The remaining 2,032 residents were included in the analytical sample for the assessment of functional and clinical characteristics and received treatments.

Of the 2,032 residents with diabetes, 730 (35.9%) had stage 3–5 CKD (stage 3: 532 [26.2%], stage 4: 131 [6.4%], stage 5: 67 [3.3%]). Residents with diabetes and stage 3–5 CKD were significantly older than those without stage 3–5 CKD (average age: 74.5 [±11.3] versus 71.6 [±12.7], *P* < 0.001). The majority of the study sample was female (57.6%), and the proportion of female was similar between residents with and without stage 3–5 CKD (Table 4). Of the most common comorbidities among diabetic residents, the prevalence of hypertension (92.9% versus 87.3%), depression (80.1% versus 75.4%), anemia (53.6% versus 41.5%), and congestive heart failure (CHF, 48.9% versus 34.9%) were significantly higher in residents with than residents without stage 3–5 CKD (all *P* < 0.05). Of the 812 patients with CHF, 11.0% used metformin and 2.8% used thiazolidine. A majority of residents with diabetes were either overweight (25.2%) or obese (34.1%). On average, residents with stage 3–5 CKD received more medications than those without stage 3–5 CKD (10.6 [±7.6] versus 9.9 [±7.7], *P* = 0.021) during the 90-day assessment window. Polypharmacy (receiving ≥9 unique medications) was common in diabetic residents diagnosed with and without stage 3–5 CKD (62.1% versus 56.7%, *P* = 0.073) (Table 4).

Of residents with stage 3–5 CKD, a majority of residents used cardiovascular drugs (74.9%). Overall, 54.9% of diabetic residents received at least 1 prescription for antidiabetic medications (OAD, GLP-1, or insulin) during the 90-day assessment period, and the proportion was not significantly different between residents with and without stage 3–5 CKD. A significantly higher proportion of residents with stage 3–5 CKD used insulin (46.4% versus 40.1%, *P* = 0.006), and a lower proportion used OAD or GLP-1 (28.4% versus 34.6%, *P* = 0.004) as compared to those without stage 3–5 CKD (Table 4). Of the 1,005 residents with 1+ glycated hemoglobin (HbA1c) test dated within 1 year since the NH admission, 63.5% had HbA1c <7%.

### 3.3. Assessment of Concordance of OAD Utilization with the NKF Guidelines and PIs

Of the 730 diabetic residents with diabetes and stage 3–5 CKD, 186 residents used the OADs included in the NKF guidelines during their 90-day stay in an NH. Of the 186 residents, 135 (72.6%) received the OADs in accordance with NKF guidelines. Resident demographic and clinical characteristics were similar between NKF-guideline concordant and nonconcordant cohorts except for ethnicity (Table 5). The NKF-guideline concordant cohort had a higher proportion of Hispanic and a lower proportion of White or black than the NKF nonconcordant cohort. Of the 186 residents, 77 (41.4%) received the medications in accordance with their respective PIs. No statistically significant differences in characteristics were found between the PI concordant and nonconcordant cohorts.

## 4. Discussion

There are limited data available regarding the characteristics and management of elderly residents with diabetes and chronic kidney disease in long-term care settings in the United States [19]. This real world study demonstrated a high prevalence of moderate to severe CKD (47%) in diabetic residents in US nursing homes during 2008 to 2011. The study also found that polypharmacy (number of unique medications ≥9 over a 90-day period) was common (62.1%) among diabetic residents diagnosed with stage 3–5 CKD. In addition, among the residents receiving OADs included in the NKF guidelines, about a third (27.6%) of patients did not receive the OADs in concordance with NKF guideline recommendations and over half (58.6%) did not receive the OADs in concordance with PIs.

Consistent with other studies, the results of our study suggested that the prevalence of CKD is high among individuals with diabetes. According to the CDC, approximately 44% of individuals with diabetes also had CKD in 2008 [1]. A study by Koro et al. also found that 40% of adults with diabetes had CKD [18]. However, their study looked at any degree of CKD, whereas the present study focused on the prevalence of stage 3–5 CKD among older residents with an average age of 75 years old. Given the high prevalence of CKD it is important that physicians consider the treatment plans for diabetics by assessing for renal impairment.

In our sample, only 18% of residents were identified as having renal insufficiency/renal failure in the MDS, whereas a much higher proportion was identified as having moderate to severe CKD based on lab tests. The results suggested that the presence of moderate to severe CKD may have been under-documented in the MDS. The MDS data collection form had a prespecified list of health conditions, including chronic renal failure (MDS 2.0) and renal insufficiency/renal failure (MDS 3.0), and nurses could have checked each applicable item based on residents' charts. Nurses may have under-documented the condition due to the lack of detailed instruction on how to define or document renal insufficiency or renal failure, or because residents' charts did not clearly specify the presence of CKD. Although there are additional fields for nurses to enter ICD-9-CM diagnostic codes, those fields are often reserved to document conditions not included in the checklist. In this study, few additional NH residents were identified as having moderate to severe CKD by scanning the ICD-9-CM codes in the MDS. The identification of CKD in NH settings needs to be improved and residents with moderate to severe CKD should be clearly flagged about their condition in the MDS, which will trigger a careful evaluation of their treatment plans.

This study identified about a third of residents who received at least one OAD not in concordance with NKF guidelines and over two-thirds not in concordance with PIs. A prior study by Chen et al. found that patients with diabetes diagnosed with moderate to severe CKD receiving OADs according to the NKF guidelines concordance showed better clinical outcomes (i.e., better glycemic control) compared with nonconcordant residents [17]. Given the association with positive clinical outcomes, increasing awareness of use of medications according to the NKF guidelines and PI concordance should be made through physician (and nurses') education.

Our findings also suggest that Hispanic NH residents with CKD were more likely to receive antidiabetic medications concordant with NKF guidelines than other residents. It is unclear whether it is because Hispanic residents received different care, or because the Hispanic residents were clustered in NHs that provided higher quality of care. A cross-sectional study of CKD patients revealed that CKD complications were more common, and mean eGFR was lower among Hispanics than Black or White patients [21], which may have triggered NH staff conduct more careful assessment of patient medication use. However, given the small sample size, the results should be interpreted with caution.

To the best of our knowledge, the results of this study provide the most recent findings on polypharmacy among the US NH residents to date. More than half of the residents with diabetes (58.6%) were recipients of polypharmacy during the 90-day assessment window, and the estimate was even higher in residents with both diabetes and moderate to severe CKD (62.1%). This proportion was much higher than the 40% reported by an NH survey in 2004 [14] and the 32% reported by the Medical Expenditure Panel Survey-Nursing Home Component (MEPS-NHC) in 1996 [20]. One probable reason is that in our study, we assessed the medication use among diabetic residents, and an abundance of literature has suggested that the diabetic elderly have more complications and comorbidities than those without diabetes [3, 8, 9]. Prior literature has shown that there is a positive association between polypharmacy, inappropriate medication use, adverse events, and health care costs [14, 22]. It is therefore important to undertake regular medication review and modify the drug regimen of NH residents with diabetes and CKD to reduce the adverse events, minimize costs, and improve the quality of NH care.

Approximately half of the residents with diabetes included in the current study received prescriptions for any antidiabetic medications which included OAD and/or insulin during a 90-day window of observation. To confirm this, we performed a sensitivity analysis in which we expanded the assessment period of medication use to 1 year to minimize the probability that residents filled prescriptions outside of the 90-day assessment window. The estimated proportion of residents with diabetes treated with antidiabetic medications only increased to slightly over 60%. The prescription data in AnalytiCare LTC data reflect all the medications dispensed during residents' NH stays. Since medication use was closely monitored in the nursing home settings (e.g., NH staff dispense and administer medications), we believe that the prescription information accurately reflect the medications being taken by the residents during their stays. The low use of antidiabetic treatments could be due to better diet control, undernutrition and low food intake (especially among the those ≥80 years old), providers' concerns of adverse events such as hypoglycemia, financial constraints in the NHs, or poor monitoring of glycemic control in the LTC settings. Furthermore, the glucose target of older NH residents with diabetes may be less stringent as compared to the target of relatively younger and healthier community-dwelling patients with diabetes. For example, Fravel et al. recommended that providers weigh the benefit and risk of strict glycemic control given the frailty and clinical complexity of elderly individuals with diabetes [10], which may explain the low use of antidiabetic medications among NH residents. Further studies are needed to assess the reasons and associated risk and benefit of low use of antidiabetic medications in the NH settings.

## 5. Study Limitations

There were some limitations in this study. The MDS data collection form had a prespecified list of health conditions for nurses to check. However, there is limited instruction on how to identify or document the presence of chronic conditions in MDS, including diabetes or renal insufficiency/renal failure. We were unable to differentiate residents with type I or type II diabetes or assess the stages of CKD diagnosis assigned to the residents using the information in MDS. The stage of CKD among diabetic patients was assessed based on one eGFR reading. Some residents may have acute kidney impairment and may be misclassified as having CKD. Furthermore, because the data were derived from a limited number of facilities located mainly in Texas and Colorado, for which information on laboratory results are available, the findings on the prevalence of CKD and pattern of medication use and concordance to PI and NKF guidelines cannot be generalized to the US population. Less than half of the residents had glycated hemoglobin measures within 1 year since the NH admission. Thus, we cannot assess the glycemic control among these residents.

## 6. Conclusion

This study provides recent insights into the prevalence of CKD and treatment patterns in elderly NH residents with diabetes. The results showed a high prevalence of moderate to severe CKD in diabetic residents. Over half of the patients received more than 9 unique medications during a 90-day assessment window. A high proportion of patients with stage 3–5 CKD did not receive OADs as per the NKF guidelines or PI. Given the complex treatment regimen for diabetes, more studies are warranted to assess quality care, treatment, and resources for NH populations with diabetes and CKD. Due to the increasing prevalence of diabetes in LTC residents, the findings of this study may have important public health implications.

## Figures and Tables

**Table 1:** Stages of CKD.

ICD-9-CM diagnosis code	CKD disease stage	Corresponding GFR (mL/min/1.73 m^2^)	Description
585.1	1	≥90 with kidney damage	Normal kidney function, but urine findings, structural abnormalities, or genetic trait points to kidney disease
585.2	2	60–89 with kidney damage	Mildly reduced kidney function, and other findings (as for stage 1) point to kidney disease
585.3	3	30–59	Moderately reduced kidney function
585.4	4	15–29	Severely reduced kidney function
585.5, 585.6	5	<15 (or dialysis)	Very severe, or end-stage kidney failure (sometimes called established renal failure)

CKD: chronic kidney disease; GFR: glomerular filtration rate; ICD-9-CM: The International Classification of Diseases, Ninth Revision, Clinical Modification.

**Table 2:** List of OADs mentioned in the NKF guidelines.

Therapeutic Class	Medication
Second-generation sulfonylureas	Glyburide, glipizide, glimepiride
Alpha-glucosidase inhibitors	Acarbose, miglitol
Biguanides	Metformin
Meglitinides	Repaglinide, nateglinide
Thiazolidinediones	Rosiglitazone, pioglitazone
DPP-4 inhibitors^‡^	Sitagliptin

DPP-4: Dipeptidyl peptidase inhibitor-4

^‡^Saxagliptin and linagliptin were not included in the NKF guideline.

They were included in the PI concordance assessment.

**Table 3:** Summary of recommendations of dosing adjustments from the NKF guidelines and PI.

Therapeutic class	Medication	Route of elimination and metabolism	NKF guidelines [15]		PIs [21-33]
			CKD stages 3, 4 or kidney transplant	Dialysis	Renal insufficiency
Second-generation sulfonylureas	Glyburide	Hepatic, with renal excretion of active metabolites	Avoid	Avoid	The initial and maintenance dosing should be conservative
	Glipizide	Hepatic, with renal excretion of active metabolites	No dosage adjustment necessary	No dosage adjustment necessary	The initial and maintenance dosing should be conservative
	Glimepiride	Hepatic, with renal excretion of active metabolites	Initiate at low dosage, 1 mg daily	Avoid	Initiate at low dosage, 1 mg daily

Alpha-glucosidase Inhibitors	Acarbose	Intestinal	Not recommended in patients with serum creatinine (SCr) > 2 mg/dL	Avoid	Not recommended in patients with SCr > 2 mg/dL
	Miglitol	Renal	Not recommended in patients with SCr > 2 mg/dL	Avoid	Not recommended in patients with SCr > 2 mg/dL

Biguanides	Metformin	Renal	Contraindicated with kidney dysfunction defined as SCr ≥1.5 mg/dL in men or ≥1.4 mg/dL in women	Avoid	Contraindicated with kidney dysfunction defined as SCr ≥1.5 mg/dL in men or ≥1.4 mg/dL in women

Meglitinides	Repaglinide	Hepatic	No dosage adjustment necessary	No dosage adjustment necessary	Initiate with 0.5 mg dose for patients with severe renal function impairment (creatinine clearance 20–40 mL/min). Not recommended in patients with creatinine clearance below 20 mL/min or hemodialysis
	Nateglinide	Hepatic, with renal excretion of active metabolites	Initiate at low dosage, 60 mg before each meal	Avoid	No dosage adjustment necessary

Thiazolidinediones	Rosiglitazone	Hepatic	No dosage adjustment necessary	No dosage adjustment necessary	No dosage adjustment necessary
	Pioglitazone	Hepatic	No dosage adjustment necessary	No dosage adjustment necessary	No dosage adjustment necessary

DPP-4 inhibitors	Sitagliptin	Primarily renal	Reduce dosage by 50% (50 mg/day) when 30 ≤ GFR < 50 mL/min/1.73 m^2^ and by 75% (25 mg/day) when GFR < 30 mL/min/1.73 m^2^	Reduce dosage by 75% (25 mg/day)	Reduce dosage to 50 mg once daily when CrCl ≥30 to <50 mL/min, approximately corresponding to serum creatinine levels of >1.7 to ≤3.0 mg/dL in men and >1.5 to ≤2.5 mg/dL in women. Reduce dosage to 25 mg once daily when CrCl <30 mL/min, approximately corresponding to serum creatinine levels of >3.0 mg/dL in men and >2.5 mg/dL in women
	Saxagliptin	Both renal and hepatic	Not reported	Not reported	Reduce dosage to 2.5 mg once daily for patients with moderate or severe renal impairment, or end-stage renal disease (CrCl ≤50 mL/min)
	Linagliptin	Nonrenal pathways	Not reported	Not reported	No dosage adjustment necessary

SD: standard deviation; NKF: National Kidney Foundation; PI: prescribing information; COPD: chronic obstructive pulmonary disease; GFR: glomerular filtration rate; CrCl: creatinine clearance.

**Table 4:** Sociodemographic and clinical characteristics of nursing home residents with diabetes, by presence of moderate to severe chronic kidney disease.

	All (*n* = 2,032)	CKD stage		
		CKD stage < 3 (*n* = 1,302)	CKD stages 3–5 (*n* = 730)	*P* value
Age (mean, SD)	72.6 (12.3)	71.6 (12.7)	74.5 (11.3)	<0.001
Female, *N* (%)	1,171 (57.6)	743 (57.1)	428 (58.6)	0.494
Ethnicity, *N* (%)				0.579
White, not of Hispanic origin	1,190 (58.6)	777 (59.7)	413 (56.6)	
Black, not of Hispanic origin	281 (13.8)	181 (13.9)	100 (13.7)	
Hispanic	501 (24.7)	306 (23.5)	195 (26.7)	
Other	60 (2.9)	38 (2.9)	22 (3.0)	
Location of facility, *N* (%)				0.408
Texas	1,156 (56.9)	754 (57.9)	402 (55.1)	
Colorado	816 (40.2)	508 (39.0)	308 (42.2)	
Other	60 (2.9)	40 (3.1)	20 (2.7)	
Health conditions, *N* (%)				
Hypertension	1,815 (89.3)	1,137 (87.3)	678 (92.9)	<0.001
Depression	1,567 (77.1)	982 (75.4)	585 (80.1)	0.015
Diabetic retinopathy	1,136 (55.9)	723 (55.5)	413 (56.6)	0.649
Dementia other than Alzheimer's	1,111 (54.7)	726 (55.8)	385 (52.7)	0.189
anemia	931 (45.8)	540 (41.5)	391 (53.6)	<0.001
Congestive heart failure	812 (40.0)	455 (34.9)	357 (48.9)	<0.001
Functional status, *N* (%)				
Activities of daily living: extensive assistance to total dependence	1,393 (68.5)	892 (68.5)	501 (68.6)	0.956
Cognitive function: moderate—very severe cognitive impairment	964 (47.5)	639 (49.1)	325 (44.6)	0.048
Body mass index, *N* (%)				0.043
Underweight (<18.5)	101 (5.0)	76 (5.8)	25 (3.4)	
Normal (18.5–25)	715 (35.2)	469 (36.0)	246 (33.7)	
Overweight (25–30)	513 (25.2)	325 (25.0)	188 (25.8)	
Obese (>30)	693 (34.1)	427 (32.8)	266 (36.4)	
Missing	10 (0.5)	5 (0.4)	5 (0.7)	
Medication use, *N* (%)				
Cardiovascular drugs	1,448 (71.3)	901 (69.2)	547 (74.9)	0.006
Diuretics drugs	747 (36.8)	440 (33.8)	307 (42.1)	<0.001
Antidepressants	1,061 (52.2)	653 (50.2)	408 (55.9)	0.013
Antipsychotic agents	500 (24.6)	348 (26.7)	152 (20.8)	0.003
Antihistamines	237 (11.7)	137 (10.5)	100 (13.7)	0.032
Opiates	793 (39.0)	503 (38.6)	290 (39.7)	0.628
Antispasmodic agents (skeleton muscle)	123 (6.1)	82 (6.3)	41 (5.6)	0.537
Antispasmodic agents (smooth muscle)	182 (9.0)	125 (9.6)	57 (7.8)	0.175
Parkinson's drug	38 (1.9)	29 (2.2)	9 (1.2)	0.112
Total number of unique medication used (mean, SD)	10.14 (7.7)	9.91 (7.7)	10.56 (7.6)	0.021
0–3, *N* (%)	494 (24.3)	325(25.0)	169 (23.2)	
4–8, *N* (%)	347 (17.1)	239 (18.4)	108 (14.8)	
9–14, *N* (%)	647 (31.8)	407 (31.3)	240 (32.9)	
≥15, *N* (%)	544 (26.8)	331 (25.4)	213 (29.2)	
Proportion with polypharmacy (9+ medications)	1,191 (58.6)	738 (56.7)	453 (62.1)	0.073
Any antidiabetic medications, *N* (%)	1,116 (54.9)	704 (54.1)	412 (56.4)	0.303
Oral antidiabetic drug or GLP-1	657 (32.3)	450 (34.6)	207 (28.4)	0.004
Oral antidiabetic drugs	612 (30.1)	426 (32.7)	186 (25.5)	<0.001
Sulfonylurea 2nd generation	303 (14.9)	193 (14.8)	110 (15.1)	0.882
Metformin	321 (15.8)	265 (20.4)	56 (7.7)	<0.001
Thiazolidinediones	84 (4.1)	54 (4.1)	30 (4.1)	0.967
Nonsulfonylurea secretagogues	9 (0.4)	3 (0.2)	6 (0.8)	0.054
Alpha glucosidase inhibitor	1 (0.0)	1 (0.1)	0 (0.0)	0.454
Dipeptidyl peptidase-4 inhibitor	34 (1.7)	16 (1.2)	18 (2.5)	0.037
Amylin analogue	0 (0.0)	0 (0.0)	0 (0.0)	N/A
Combination of oral antidiabetic medications	18 (0.9)	11 (0.8)	7 (1.0)	0.792
GLP or GLP-1 injectables	69 (3.4)	39 (3.0)	30 (4.1)	0.183
Insulin	861 (42.4)	522 (40.1)	339 (46.4)	0.006

HbA1c reading dated within 1 year since NH admission	*n* = 1,005	*n* = 667	*n* = 338	0.267
HbA1c < 7%	638 (63.5)	436 (65.4)	202 (59.8)	
7 ≤ HbA1c < 8%	174 (17.3)	109 (16.3)	65 (19.2)	
7 ≤ HbA1c < 9%	117 (11.6)	77 (11.5)	40 (11.8)	
HbA1c ≥ 9%	76 (7.6)	45 (6.8)	31 (9.2)	

CKD: chronic kidney disease; SD: standard deviation; GLP-1: glucagon-like peptide-1 agonists; HbA1c: glycated hemoglobin; eGFR: estimated glomerular filtration rate.

**Table 5:** Sociodemographic and clinical characteristics of residents receiving selected OAD.

	All	NKF guideline			PI recommendations		
	(*n* = 186)	Nonconcordant (*n* = 51)	Concordant (*n* = 135)	*P* value	Nonconcordant (*n* = 109)	Concordant (*n* = 77)	*P* value
	*N* (%)	*N* (%)	*N* (%)		*N* (%)	*N* (%)	
Age (mean, SD)	75.0 (11.8)	73.2 (14.1)	75.7 (10.8)	0.2572	74.7 (12.4)	75.5 (11.1)	0.6491
Female	118 (63.4)	30 (58.8)	88 (65.2)	0.4216	65 (59.6)	53 (68.8)	0.1995
Ethnicity				0.0499			0.8313
White (not of Hispanic origin)	104 (55.9)	32 (62.7)	72 (53.3)		62 (56.9)	42 (54.5)
Black (not of Hispanic origin)	26 (14.0)	10 (19.6)	16 (11.9)		17 (15.6)	9 (11.7)
Hispanic	51 (27.4)	7 (13.7)	44 (32.6)		28 (25.7)	23 (29.9)
Asian/pacific islanders	2 (1.1)	0 (0.0)	2 (1.5)		1 (0.9)	1 (1.3)
Other	3 (1.6)	2 (3.9)	1 (0.7)		1 (0.9)	2 (2.6)
Facility state				0.6940			0.9357
Colorado	76 (40.9)	22 (43.1)	54 (40.0)		43 (39.4)	33 (42.9)	
Texas	103 (55.4)	26 (51.0)	77 (57.0)		61 (56.0)	42 (54.5)	
Other	7 (3.8)	3 (5.9)	4 (3.0)		5 (4.6)	2 (2.6)	

SD: standard deviation; NKF: National Kidney Foundation; PI: prescribing information; COPD: chronic obstructive pulmonary disease.
